# Post-Translational Protein Deimination Signatures in Serum and Serum-Extracellular Vesicles of *Bos taurus* Reveal Immune, Anti-Pathogenic, Anti-Viral, Metabolic and Cancer-Related Pathways for Deimination

**DOI:** 10.3390/ijms21082861

**Published:** 2020-04-19

**Authors:** Michael F. Criscitiello, Igor Kraev, Sigrun Lange

**Affiliations:** 1 Comparative Immunogenetics Laboratory, Department of Veterinary Pathobiology, College of Veterinary Medicine and Biomedical Sciences, Texas A&M University, College Station, TX 77843, USA; mcriscitiello@cvm.tamu.edu; 2 Department of Microbial Pathogenesis and Immunology, College of Medicine, Texas A&M Health Science Center, Texas A&M University, College Station, TX 77843, USA; 3 Electron Microscopy Suite, Faculty of Science, Technology, Engineering and Mathematics, Open University, Milton Keynes MK7 6AA, UK; igor.kraev@open.ac.uk; 4 Tissue Architecture and Regeneration Research Group, School of Life Sciences, University of Westminster, London W1W 6XH, UK

**Keywords:** peptidylarginine deiminases (PADs), protein deimination, bovine (*Bos taurus*), extracellular vesicles (EVs), immunity, metabolism, anti-viral

## Abstract

The bovine immune system is known for its unusual traits relating to immunoglobulin and antiviral responses. Peptidylarginine deiminases (PADs) are phylogenetically conserved enzymes that cause post-translational deimination, contributing to protein moonlighting in health and disease. PADs also regulate extracellular vesicle (EV) release, forming a critical part of cellular communication. As PAD-mediated mechanisms in bovine immunology and physiology remain to be investigated, this study profiled deimination signatures in serum and serum-EVs in *Bos taurus*. *Bos* EVs were poly-dispersed in a 70–500 nm size range and showed differences in deiminated protein cargo, compared with whole sera. Key immune, metabolic and gene regulatory proteins were identified to be post-translationally deiminated with some overlapping hits in sera and EVs (e.g., immunoglobulins), while some were unique to either serum or serum-EVs (e.g., histones). Protein–protein interaction network analysis of deiminated proteins revealed KEGG pathways common for serum and serum-EVs, including complement and coagulation cascades, viral infection (enveloped viruses), viral myocarditis, bacterial and parasitic infections, autoimmune disease, immunodeficiency intestinal IgA production, B-cell receptor signalling, natural killer cell mediated cytotoxicity, platelet activation and hematopoiesis, alongside metabolic pathways including ferroptosis, vitamin digestion and absorption, cholesterol metabolism and mineral absorption. KEGG pathways specific to EVs related to HIF-1 signalling, oestrogen signalling and biosynthesis of amino acids. KEGG pathways specific for serum only, related to Epstein–Barr virus infection, transcription mis-regulation in cancer, bladder cancer, Rap1 signalling pathway, calcium signalling pathway and ECM-receptor interaction. This indicates differences in physiological and pathological pathways for deiminated proteins in serum-EVs, compared with serum. Our findings may shed light on pathways underlying a number of pathological and anti-pathogenic (viral, bacterial, parasitic) pathways, with putative translatable value to human pathologies, zoonotic diseases and development of therapies for infections, including anti-viral therapies.

## 1. Introduction

Cattle are mammalian ruminants of the genus *Bos*, comprised of domesticated and wild cattle with five main extant (living) species [1]. The lifespan of *Bos* is 18-25 years in the wild and as cattle are valuable livestock that form an important part of food security, bovine research is important for livestock management. Furthermore cows fall under a group of long-lived mammals that display considerable cancer resistance [2]. With considerably long life spans and unusual immunological characteristics cows may hold information for molecular pathways underlying such physiological traits. The bovine immune system has received considerable attention in the medical field due to its unique immunoglobulin traits, including exceptional ability to reach recessed viral epitopes on enveloped viruses. Therefore, a particular research focus has been on their unusual ultralong CDR3H “cattlebodies”, which are being developed for immunotherapy, including against retroviral infections such as HIV [3,4,5].

Peptidylarginine deiminases (PADs) are phylogenetically conserved calcium-dependent enzymes which cause an irreversible post-translational conversion of arginine to citrulline in target proteins. This modification causes structural, and sometimes functional, changes of target cytoskeletal, cytoplasmic, mitochondrial and nuclear proteins, including loss or gain of function or denaturation. Deimination can furthermore cause the generation of neo-epitopes and affect gene regulation [6,7,8,9,10,11]. This post-translational modification is most effective on beta-sheets and disordered proteins [7] and can also facilitate protein moonlighting, where one polypeptide can exhibit multifaceted functions that are physiologically relevant. As this is an evolutionarily acquired phenomenon, moonlighting facilitated by post-translational changes, such as deimination, may contribute to protein’s diverse and conserved functions throughout phylogeny [12,13]. PADs are identified throughout phylogeny from bacteria to mammals. In mammals, five tissue specific PAD isozymes with deimination activity are described, three in chicken and alligator, one in bony and cartilaginous fish [6,14,15,16,17], and PAD homologues (arginine deiminases, ADI) in parasites [18], fungi [19] and bacteria [20,21]. While in *Bos taurus* five PAD isozymes have been reported: PAD1 (NP_001094742.1), PAD2 (NP_001098922.1), PAD3 (XP_010800991.1), PAD4 (NP_001179102.1) and PAD6 (XP_002685843.1), few studies, besides assessment of cattle PAD ability to deiminate human myelin basic protein [22] and inhibitory effects of paclitaxel on PAD activity in bovine brain extract [23], have hitherto been carried out on PAD protein function, or on putative physiological relevance for PAD-mediated post-translational deimination in cattle.

PADs play important roles in a range of pathologies, including chronic, autoimmune and neurodegenerative diseases, as well as in cancer [9,10,11,24]. PADs also play roles in hypoxia and CNS regeneration [25,26,27,28,29], and PAD-mediated mechanisms have been related to ageing [30,31]. Importantly, PADs have also been implicated in infection, including sepsis, endotoxemia [32,33,34,35,36,37,38,39], in antibiotic resistance [21] and other anti-pathogenic responses, including anti-viral ones [37,40,41]. Roles for anti-viral responses via PAD-mediated neutrophil extracellular trap formation (NETosis) have furthermore been identified in cattle respiratory syncytial virus disease, via the detection of deiminated/citrullinated histone H3 [42]. Roles for PADs in tissue remodeling and immunity have also recently been described [15,16,43]. PADs have furthermore been identified as phylogenetically conserved key regulators of cellular extracellular vesicle (EV) release [21,44,45,46]. EV-mediated cellular communication is a phylogenetically conserved phenomenon [47], with EVs transferring cargo proteins and genetic material characteristic of the cells of origin [11,48,49,50,51]. As EV cargo is comprised of a large range of proteins, enzymes and genetic material, and as EVs can easily be isolated from a range of body fluids, including serum and plasma, EV signatures can be useful biomarkers [52,53]. 

While work on EVs has largely focused on human pathologies [53], EVs are gaining increasing interest also in veterinary medicine, including in cattle [47,54,55,56,57]. Studies on EVs in cattle have been in relation to host-pathogen interactions [58,59,60,61], including in bovine respiratory disease [62,63] and in response to infection [64,65], as well as for export of viral proteins [66,67,68]. EV research in cattle has furthermore been in relation to the estrous cycle, fertility and reproduction [56,69,70,71,72,73,74], as well as for roles during embryonic development [75,76,77,78]. Bovine milk EVs have been assessed for biological activities [79,80,81,82,83], for the application of milk EVs as safe drug delivery vehicles [84,85,86], as well as assessment of viral transfer via milk to calves [87]. Bovine milk EVs have also been investigated as an anti-inflammatory treatment in autoimmune disease [88,89] and in necrotizing enterocolitis [90]. Cattle EVs have furthermore been investigated in relation to neurological disease, including bovine spongiform encephalopathy (BSE) [54]. 

A recent body of comparative studies with respect to EVs and EV cargo has been performed in a range of taxa throughout the phylogenetic tree, including with a particular emphasis on deiminated protein cargo by our group [17,91,92,93,94,95,96,97,98,99] Hitherto though, no such evaluation of post-translational protein cargo in cattle serum or serum-EVs has been carried out and therefore warrants further exploration. In the current study, post-translationally deiminated protein signatures were assessed in serum and serum-EVs of *Bos taurus*. We report for the first time post-translational deimination of key immune, metabolic and nuclear proteins in cattle, and identify differences in KEGG pathways enriched for deiminated proteins in serum-EVs compared to whole serum. Our findings provide novel insight into the unusual immunological traits of cattle, including a new angle on their unusual anti-viral activity. Our findings further current understanding of protein moonlighting via deimination in physiological and immunological pathways, underlying anti-pathogenic responses throughout phylogeny.

## 2. Results

### 2.1. Characterisation of Bovine Serum-EVs 

**Figure 1:**
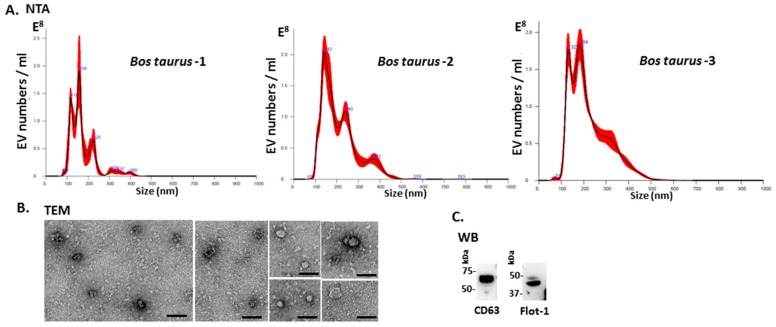
Extracellular vesicle profiling in bovine serum. (**A**) Nanoparticle tracking analysis shows a size distribution of plasma-EVs from *Bos taurus* in the size range of 70 to 500 nm, with main peaks at approximately 120–240 nm. (**B**) Transmission electron microscopy (TEM) analysis of bovine serum-derived EVs shows typical EV morphology; scale bar is 50 nm in all figures. (**C**) Western blotting analysis confirms that bovine EVs are positive for the phylogenetically conserved EV-specific markers CD63 and Flot-1, showing positive at expected molecular weight size corresponding to what is observed in other taxa (kDa = kilodaltons).

*Bos taurus* serum-EVs were assessed by nanoparticle tracking analysis (NTA) for particle numbers and size distribution using the NanoSight NS300 system, revealing a poly-dispersed population of EVs in the size range of 70–500 nm (Figure 1A). Further characterization was performed by western blotting using the EV-specific markers CD63 and Flot-1 (Figure 1B), which showed positive at protein band sizes corresponding to what is observed for these EV markers in other taxa, including in human [17,46,91,92,93,96,97,98,99], and by transmission electron microscopy (TEM), confirming typical EV morphology (Figure 1C). Some variation was observed between the three individuals with respect to EV yield, ranging from 1.72 × 10^10^ to 2.46 × 10^10^ per mL, and modal EV size, which fell in the range of 146–165 nm. 

### 2.2. PAD Protein Homologues and Deiminated Proteins in Bovine Serum and Serum-EVs

**Figure 2:**
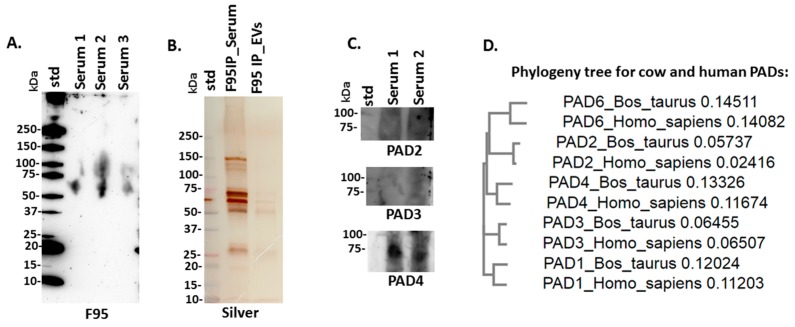
Deiminated proteins and peptidylarginine deiminases (PADs) in bovine serum. (**A**) Total deiminated proteins were identified in bovine serum using the pan-deimination specific F95 antibody. (**B**) F95-enriched IP fraction from bovine serum and serum-extracellular vesicles (EVs), shown by silver-staining. (**C**) Immunodetection of PAD homologues in bovine sera by western blotting, using anti-human PAD2, PAD3 and PAD4 antibodies. (**D**) A neighbor-joining phylogeny tree for bovine and human PAD isozymes.

For assessment of total deiminated proteins present in serum, F95-positive proteins were detected by western blotting in the size range of mainly 50–150 kDa (Figure 2A). Following immunoprecipitation for F95-enrichment of deiminated proteins in serum and serum-EVs, silverstaining revealed F95-enriched protein bands between 15–150 kDa in serum and in EVs protein bands were observed mainly in the size range of 50–150 kDa (Figure 2B). For assessment of bovine PAD protein homologues, anti-human PAD-isozyme specific antibodies were used for western blotting, identifying a positive protein band at 70–75 kDa mainly cross-reacting with anti-human PAD4 antibody in bovine serum (Figure 2C). A neighbor-joining phylogeny tree was furthermore constructed for bovine PADs (PAD1, NP_001094742.1; PAD2, NP_001098922.1; PAD3, XP_010800991.1; PAD4, NP_001179102.1 and PAD6, XP_002685843.1) compared with human PADs (PAD1, NP_037490.2; PAD2, NP_031391.2; PAD3, NP_057317.2; PAD4, NP_036519.2 and PAD6, NP_997304.3), using Clustal Omega (https://www.ebi.ac.uk/Tools/msa/clustalo/), showing that the individual bovine and human PAD isoforms clustered together (Figure 2D). 

### 2.3. LC-MS/MS Analysis of Deiminated Proteins in Bovine Serum and Serum-EVs

Protein identification of deiminated proteins in bovine serum and serum-EVs was carried out using F95-enrichment and LC-MS/MS analysis, searching for species-specific protein hits using the *Bos taurus* protein database. In serum, 118 species-specific deiminated protein hits were identified and 31 were specific to total serum only (Table 1; see detailed analysis for all hits in Supplementary Table S1). In serum-EVs, 179 species-specific deiminated protein hits were identified and 90 were specific to EVs only (Table 2; see detailed analysis for all hits in Supplementary Table S2). 

**Figure 3:**
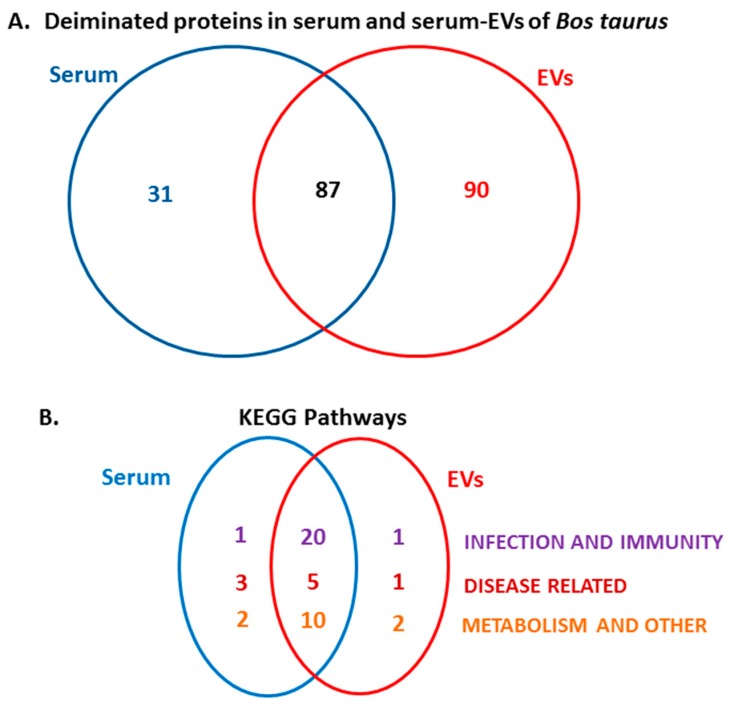
Deiminated proteins identified in bovine serum and serum-EVs. (**A**) Species specific hits identified for deiminated proteins in bovine serum (Table 1) and serum-EVs (Table 2) respectively, as well as number of overlapping hits are presented. (**B**) KEGG (Kyoto Encyclopedia of Genes and Genomes) pathways identified to be enriched in deiminated proteins in bovine serum and serum-EVs respectively, as well as number of overlapping KEGG pathways, are presented. For specific KEGG pathways presented in the Venn diagram, see the protein–protein interaction networks in Figure 4, Figure 5, Figure 6, Figure 7 and Figure 8.

The Venn diagram in Figure 3 represents common and specific deimination hits in serum and serum-EVs. Overall 87 deiminated protein hits were common to serum and serum-EVs, while 31 hits were identified to be deiminated in serum only and 90 hits were identified to be deiminated in EVs only (Figure 3A). Following KEGG pathway analysis for these deiminated protein hits, a number of common KEGG pathways enriched in deiminated proteins were identified in serum and serum EVs, while differences were observed in KEGG pathways relating to infection, immunity, disease and metabolism, that were specific to serum or serum-EVs only, respectively (Figure 3B). 

### 2.4. Protein–Protein Interaction Network Identification of Deiminated Proteins in Bovine Serum and Serum-EVs

**Figure 4:**
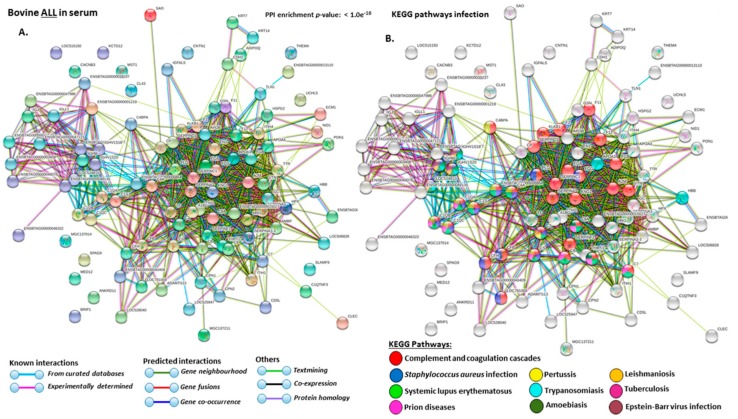

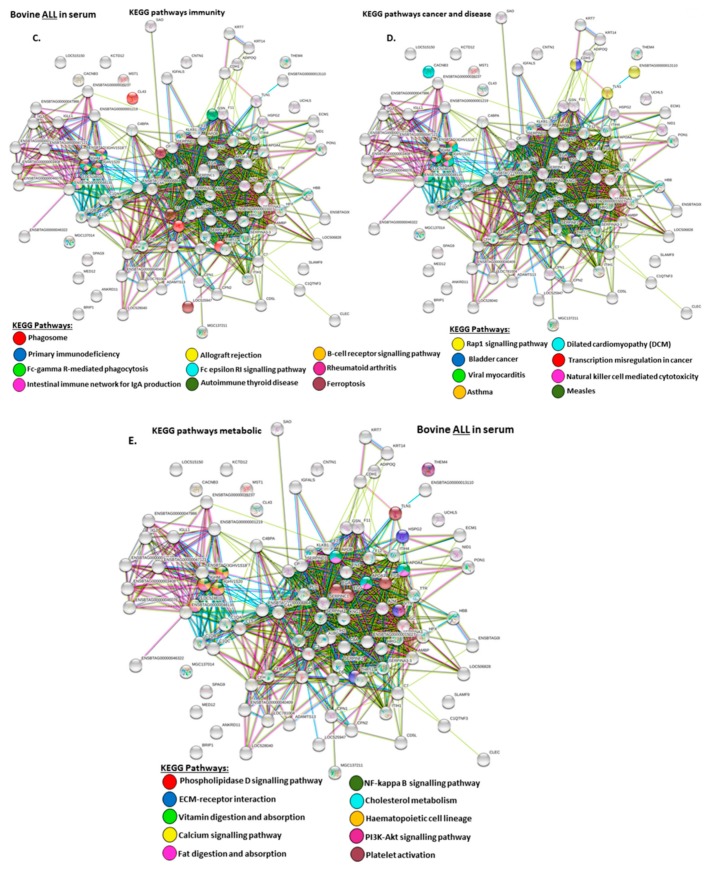
Protein–protein interaction networks show all deiminated proteins which were identified in bovine serum. Protein–protein interactions were reconstructed based on both known and predicted interactions in serum of *Bos taurus*, using STRING analysis. (**A**) Query proteins are indicated by the coloured nodes represent and first shell of interactors. (**B**) KEGG pathways relating to the identified proteins and reported in STRING and relating to infection are highlighted (see colour code included in the figure). (**C**). KEGG pathways relating to the identified proteins and reported in STRING are highlighted for immunity (see colour code included in the figure). (**D**). KEGG pathways relating to cancer and disease for deiminated proteins identified are highlighted (see colour code included in the figure). (**E**). KEEG pathways relating to metabolism for deiminated proteins identified are highlighted (see the colour code included in the figure). The coloured lines highlight which protein interactions are identified through known interactions (this refers to curated databases, experimentally determined), through predicted interactions (this refers to gene neighborhood, gene fusion, gene co-occurrence) or through co-expression, text mining or protein homology (the colour key for connective lines is included in the figure).

**Figure 5:**
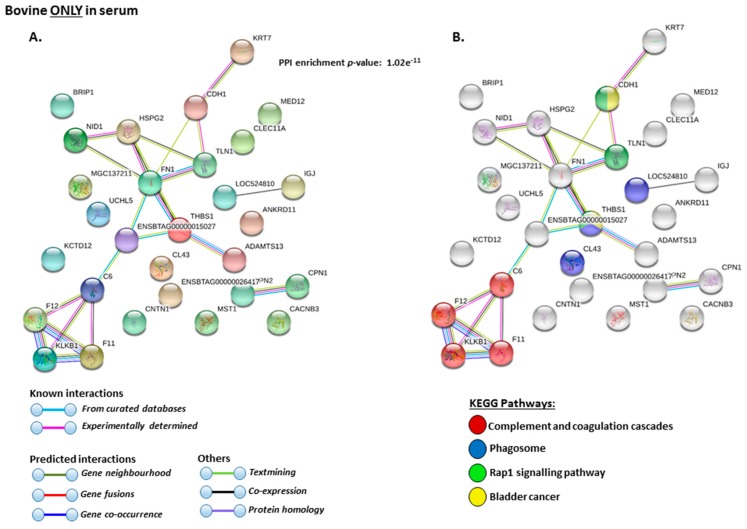
Protein–protein interaction networks of deiminated protein candidates identified in bovine serum only (not identified in EVs). Protein–protein interactions were reconstructed based on both known and predicted interactions by STRING analysis. (**A**) Query proteins are indicated by the coloured nodes and represent the first shell of interactors. (**B**) KEGG pathways reported in STRING and related to the identified proteins are highlighted (see the colour code included in the figure). The coloured lines highlight which protein interactions are identified through known interactions (this refers to curated databases, experimentally determined), through predicted interactions (this refers to gene neighborhood, gene fusion, gene co-occurrence) or through co-expression, text mining or protein homology (the colour key for connective lines is included in the figure).

**Figure 6:**
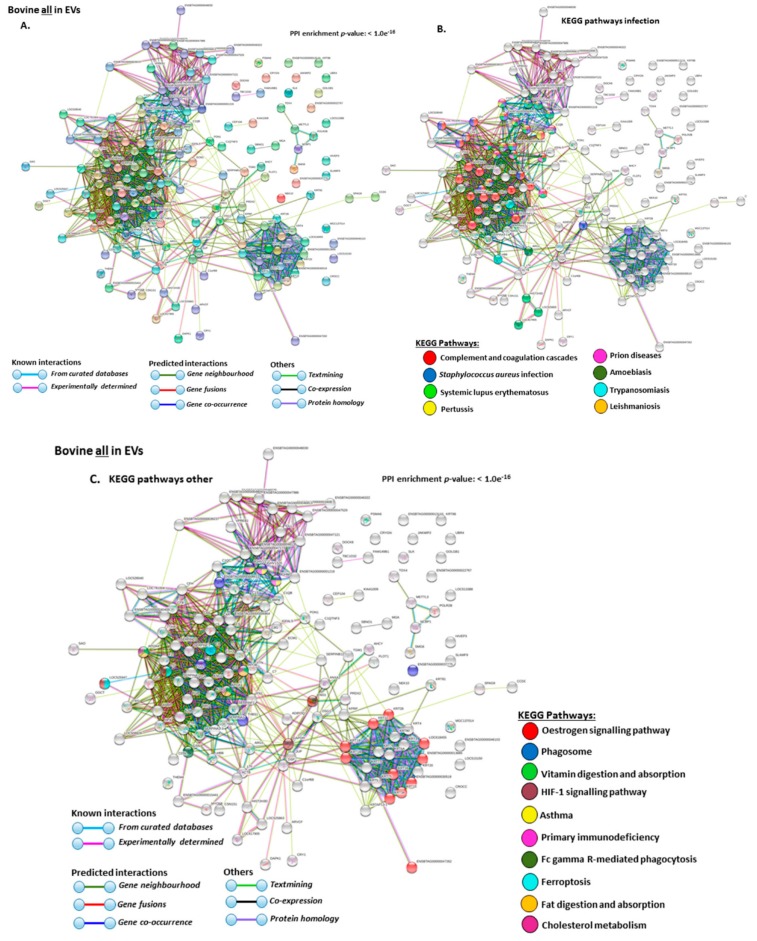
Protein–protein interaction networks of all deiminated proteins identified in serum-EVs of *Bos taurus*. Protein–protein interactions were reconstructed based on both known and predicted interactions by STRING analysis. (**A**) Query proteins are indicated by the coloured nodes and represent the first shell of interactors. (**B**) KEGG pathways reported in STRING and relating to infection are highlighted (see colour code included in the figure). (**C**) KEGG pathways relating to the identified proteins and reported in STRING are highlighted for other pathways (see colour code included in the figure). The coloured lines highlight which protein interactions are identified through known interactions (this refers to curated databases, experimentally determined), through predicted interactions (this refers to gene neighborhood, gene fusion, gene co-occurrence) or through co-expression, text mining or protein homology (the colour key for connective lines is included in the figure).

**Figure 7:**
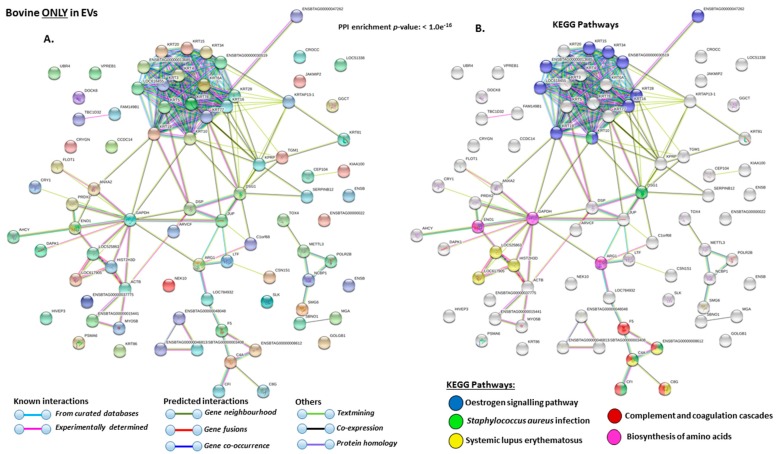
Protein–protein interaction networks of deiminated protein candidates identified in bovine serum-EVs only (not identified in total serum). Protein–protein interactions were reconstructed based on both known and predicted interactions by STRING analysis. (**A**) Query proteins are indicated by the coloured nodes and represent the first shell of interactors. (**B**) KEEG pathways relating to the identified deiminated proteins and reported in STRING are highlighted (see colour code included in the figure). The coloured lines highlight which protein interactions are identified through known interactions (this refers to curated databases, experimentally determined), through predicted interactions (this refers to gene neighborhood, gene fusion, gene co-occurrence) or through co-expression, text mining or protein homology (the colour key for connective lines is included in the figure).

**Figure 8:**
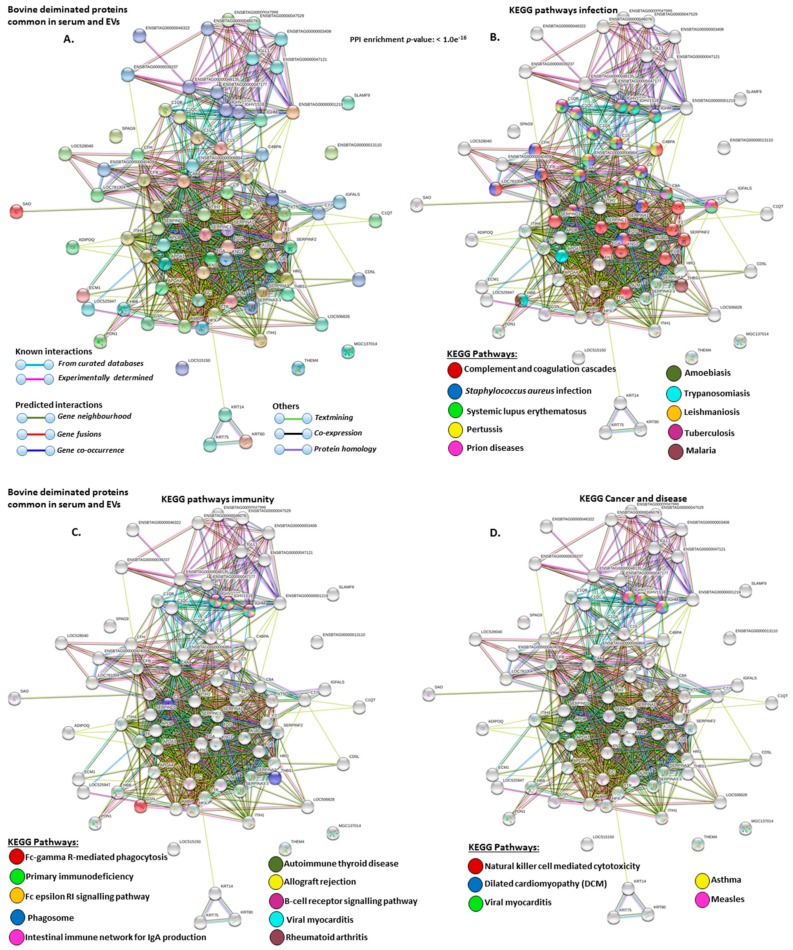

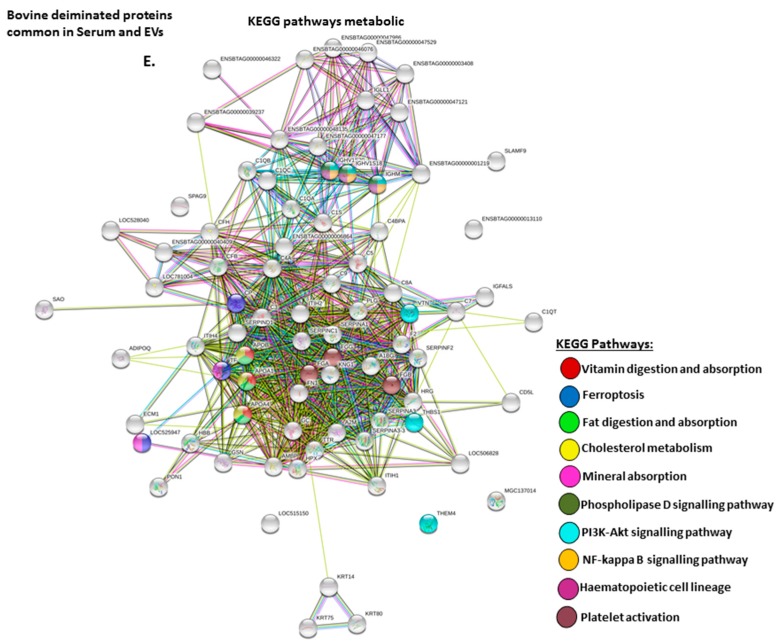
Protein–protein interaction networks for common deiminated protein candidates identified in bovine serum and serum-EVs (excluding serum-specific or EV specific candidates). Protein–protein interactions were reconstructed based on both known and predicted interactions by STRING analysis. (**A**) Query proteins are indicated by the coloured nodes and represent the first shell of interactors. (**B**) KEGG pathways for to the identified deiminated proteins and reported in STRING and relating to infection are highlighted (see colour code included in the figure). (**C**) KEGG pathways relating to the identified proteins and reported in STRING are highlighted for immunity (see colour code included in the figure). (**D**) KEGG pathways relating to cancer and disease for deiminated proteins identified are highlighted for (see colour code included in the figure). (**E**) KEEG pathways relating to metabolism for deiminated proteins identified are highlighted (see colour code included in the figure). The coloured lines highlight which protein interactions are identified through known interactions (this refers to curated databases, experimentally determined), through predicted interactions (this refers to gene neighborhood, gene fusion, gene co-occurrence) or through co-expression, text mining or protein homology (the colour key for connective lines is included in the figure).

For the prediction of protein–protein interaction networks of the deimination candidate proteins, the protein ID lists for serum and serum-EVs respectively, were submitted to STRING (Search Tool for the Retrieval of Interacting Genes/Proteins) analysis (https://string-db.org/) and analyzed for KEGG (Kyoto Encyclopedia of Genes and Genomes) pathways (Figure 3B and Figure 4, Figure 5, Figure 6, Figure 7 and Figure 8). Protein interaction networks were based on known and predicted interactions and represent all deiminated proteins identified in serum (Figure 4), all deiminated proteins identified in EVs (Figure 5) as well as deiminated proteins identified in serum only (Figure 6) or in EVs only (Figure 7), or common deimination candidates in EVs and serum (Figure 8). The PPI enrichment *p*-value for all deiminated proteins identified in bovine serum (based on protein identifier sequences) was found to be *p* < 1.0 × 10^−16^ and for all deiminated proteins identified in the serum-derived EVs, the PPI enrichment p-value was also found to be *p* < 1.0 × 10^−16^ (Figure 4 and Figure 5). For deiminated proteins identified in serum only (but not EVs) the PPI enrichment p-value was also *p* < 1.0 × 10^−16^ (Figure 6), while for deiminated proteins identified specifically in EVs, the PPI enrichment p-value was *p* = 4.83 × 10^−8^ (Figure 7). For common proteins found deiminated in serum and in serum-EVs, the PPI enrichment *p*-value was *p* < 1.0 × 10^−16^ (Figure 8). This indicates that in all cases, the identified protein networks have significantly more interactions than what would be expected for a random set of proteins of similar size, based on information drawn from the genome. 

The KEEG pathways related to all deiminated proteins identified as deiminated in whole serum are shown in Figure 4A–E. For KEGG pathways relating to infection these belonged to: “Complement and coagulation cascades”, “*S. aureus* infection”, “systemic lupus erythematous”, “prion diseases”, “pertussis”, “trypanosomiasis”, “amoebiasis”, “leishmaniosis”, “tuberculosis”, “Epstein–Barr virus infection” (Figure 4B); For KEGG pathways relating to immunity these related to: “phagosome”, “primary immunodeficiency”, “FC-gamma R-mediated phagocytosis”, “intestinal immune network for IgA production”, “allograft rejection”, “Fc epsilon RI signalling pathway”, “autoimmune thyroid disease”, “B-cell receptor signalling pathway”, “rheumatoid arthritis”, “ferroptosis” (Figure 4C); For KEGG pathways relating to cancer and disease these related to: “transcription misregulation in cancer”, “bladder cancer”, “viral myocarditis”, “Rap1 signalling pathway”, “natural killer cell mediated cytotoxicity”, “measles”, “dilated cardiomyopathy”, “asthma” (Figure 4D); For KEGG metabolic pathways these related to: “phospholipidase D signalling pathway”; “ECM-receptor interaction”; “vitamin digestion and absorpition”, “calcium signalling pathway”, “fat digestion and absorption”, “NF-kappa B signalling pathway”, “cholesterol catabolism”, “hematopoietic cell lineage”, “PI3K-Akt signalling pathway” and “platelet activation”(Figure 4E).

The KEEG pathways related to deiminated proteins identified as deiminated in whole serum only are shown in Figure 5A,B. These were: “complement and coagulation cascades”, “phagosome”, “Rap1 signalling pathway” and “bladder cancer”.

The KEEG pathways related to all deiminated proteins identified in EVs are shown in Figure 6A–C. For KEGG pathways relating to infection these belonged to: “complement and coagulation cascades”, “*S. aureus* infection”, “systemic lupus erythematous”, “pertussis”, “prion diseases”, “amoebiasis”, “trypanosomiasis”, “leishmaniosis” (Figure 6B); Other KEGG pathways identified were: “oestrogen signalling pathway”, “phagosome” “vitamin digestion and absorption”, “asthma”, “primary immunodeficiency”, “Fc gamma R-mediated phagocytosis”, “ferroptosis”, “ fat digestion and absorption”, “cholesterol metabolism”, “HIF-1 signalling pathway”.

The KEEG pathways related to deiminated proteins identified as deiminated in EVs only are shown in Figure 7A,B. These were: “oestrogen signalling pathway”, “*S. aureus* infection”, “complement and coagulation cascades”, “biosynthesis of amino acids”.

Deiminated proteins that were common to serum and serum-EVs were furthermore analyzed for KEGG pathways (Figure 8A–E). Pathways identified for infection were: “complement and coagulation cascades”, “*S. aureus* infection”, “systemic lupus erythematous”, “pertussis”, “prion diseases”, “amoebiasis”, “trypanosomiasis”, “leishmaniosis”, “tuberculosis”, “malaria” (Figure 8B); KEGG pathways for immunity (and diseases) were: “Fc gamma R-mediated phagocytosis”, “primary immunodeficiency”, “Fc epsilon RI signalling pathway”, “intestinal immune network for IgA production”, “autoimmune thyroid disease”, “allograft rejection”, B-cell receptor signalling pathway”, “phagosome”, ”viral myocarditis”, rheumatoid arthritis (Figure 8C); KEGG pathways for cancer and disease were: “natural killer cell mediated cytotoxicity”, “dilated cardiomyopathy (DCM)”, “viral myocarditis”, “asthma”, “measles” (Figure 8D); KEGG metabolic pathways for common deiminated proteins in serum and serum-EVs were: “Vitamin digestion and absorption”, “ferroptosis”, “fat digestion and absorption”, “cholesterol metabolism”, “mineral absorption”, “phospholipase D signalling pathway”, PI3K-Akt signalling pathway”, NF-kappa B signalling pathway”, “hematopoietic cell lineage” and “platelet activation” (Figure 8E).

## 3. Discussion

The current study is the first to profile deiminated protein signatures in serum and serum-EVs of cattle, using *Bos taurus* as a model species. F95-enrichment for deiminated proteins from serum and serum-EVs revealed a range of immunological, metabolic and gene regulatory proteins as candidates for this post-translational modification, therefore indicating hitherto under-recognized modes for protein-moonlighting of these proteins in bovine immunity and physiology. Few studies have hitherto assessed roles for PADs and deimination in cattle [22,23,42], while a range of studies have been carried out on EVs in relation to cattle immunity, fertility and development [54,57,60,61,63,65,68,74,78,83,85,88,90]. Export of post-translationally modified proteins, such as deiminated proteins in the current study, has not been assessed before in cattle serum. 

PAD proteins were assessed in bovine serum via cross-reaction to antibodies raised against human PAD2, PAD3 and PAD4 isozymes, with the strongest cross-reaction found with anti-human PAD4 at a predicted size of 70–75 kDa for PAD proteins. Protein sequence alignment of cow and human PADs showed that the individual isozymes clustered together between the two species, therefore the lack of cross-reaction with anti-human PAD2 and PAD3 antibodies may possibly be explained by differences in folding of the PADs, but will remain to be further investigated. It must be noted that neither anti-human PAD1 nor PAD6 antibodies were tested against bovine sera in the current study. The anti-human antibodies have previously been shown to cross-react with PADs from diverse taxa, with PAD2, which is considered the phylogenetically most conserved isoform, predominantly cross-reacting best with other species, including bony and cartilaginous fish, birds, mole-rat, alligator, camelids, cetaceans and pinnipeds [15,16,17,92,93,96,97,98,99]. 

A number of bovine species-specific deiminated protein candidates were identified in both serum and serum-EVs, using F95-enrichment in tandem with LC-MS/MS analysis. This analysis revealed some key metabolic and immune related proteins, with 87 characterised common deiminated proteins in serum and EVs, while 31 characterised deiminated protein hits were specific for serum and 90 characterised deiminated protein hits were specific for serum-EVs. Overall, the hit for deiminated proteins was higher in the serum-EVs, with a total hit of 179, compared to 118 in serum. Upon assessment of protein–protein interaction networks using STRING analysis, the PPI enrichment p-value for all deiminated proteins identified in bovine serum and serum-EVs, as well as for deiminated proteins identified either in serum or EVs only, indicated that the identified protein networks have significantly more interactions than expected for a random set of proteins of similar size, drawn from the genome, and that the proteins are at least partially biologically connected, as a group (Figure 4, Figure 5, Figure 6, Figure 7 and Figure 8). When assessing KEGG pathways for deiminated proteins relating to infection and immunity, diseases and metabolism, a number of pathways overlapped between serum and serum-EVs, while KEGG pathways only found in EVs related to HIF-1 signalling, oestrogen signalling and biosynthesis of amino acids. KEGG pathways specific for serum only related to Epstein–Barr virus infection, transcription misregulation in cancer, bladder cancer, Rap1 signalling pathway, calcium signalling pathway, ECM-receptor interaction. This indicates differences in cell-communication via export of deiminated proteins in serum-EVs (Figure 3B).

KEGG pathways in common with both serum and serum-EVs related to a number of immunological pathways including the complement and coagulation cascade, bacterial infection, parasitic infection and importantly viral infections, as well as viral myocarditis. The role for deimination of immunoglobulins may be of considerable importance, particularly in relation to enveloped viruses as identified in the current study via deiminated KEGG pathways highlighted for Epstein–Barr virus in cow serum. Deimination in this KEGG pathway has also recently been identified in aggressive glioblastoma cells [100], and the relationship between viral infection cancers still remains to be fully understood [101,102]. In the cow, a link between viral infections and cancer has also been made [103,104,105]. Furthermore, oncolytic viruses from cow, such as bovine herpesvirus type 1, have been considered for application as a human broad spectrum cancer therapeutic [106]. The cow is known for its unusual immune responses and is a model organism for studying neutralizing antibodies against viral infections, including HIV [3,4,5]. Understanding of immune responses against enveloped viruses is of pivotal importance as indeed coronaviruses (CoVs) including severe acute respiratory syndrome (SARS) and COVID-19, also fall under enveloped viruses. CoVs are enveloped, positive-stranded RNA viruses with a nucleocapsid, and the structure of the envelope has a crucial role in virus pathogenicity as it promotes viral assembly and release [107]. While a range of studies has focused on modelling the envelope proteins, post-translational deimination has not yet been investigated in relation to SARS and CoVs, while it has been previously identified to play roles in rhinovirus infection [41]. Therefore, the roles for post-translational modifications via deimination may play some roles in structural interactions with the virus, particularly as cow immunoglobulins were here identified as deimination candidates, both in serum as well as in serum-EVs, and cow antibodies with an extended knob structure formed from the third complementarity determining region of the heavy chain are known to bind bovine and human pathogens and be capable of remarkably broad viral neutralization [3,4,5,108]. Post-translational deimination of cow Ig’s, which are reported in the current study for the first time, have yet to be further explored. Roles for PADs in anti-viral responses have previously been identified via the generation of NETosis [37], which can be PAD-mediated and is also a recognized mechanism in anti-pathogenic functions in cattle [109]. Deimination has also been identified to modulate chemokines in anti-HIV responses [40] and higher levels of anti-cyclic citrullinated peptide antibodies have been found sera of HIV patients [110]. Higher amounts of cyclic citrullinated peptides in sera have indeed been related to infectious diseases including a number of viral, bacterial and parasitic infections [111]. The ability of PAD homologues in bacteria, for example bacterial arginine deiminase in *Mycoplasma arginini*, a Gram-negative bacterium, has furthermore been shown to act as an effective anti-viral agent against HIV [112]. Interestingly, a recent comparison of transcriptomics data between healthy and SARS-CoV-2 infected patients’ lung biopsies (https://www.ncbi.nlm.nih.gov/bioproject/PRJNA615032), reveals a 6-fold downregulation of PAD4 mRNA level, assessing 17 SARS-CoV-2 versus 106 healthy individuals. This highlights importance for PADs in virus host-pathogen interactions, including in COVID-19. Only PAD4 was reported in this COVID-19 patient cohort. Importantly, as PAD4 is involved in gene regulation and also considered one key-driver of NETosis [37], reduced PAD4 levels, observed in these COVID-19 patients, may contribute to less defences against viral infection due to gene regulatory changes, changes in deimination of immune related proteins, or impaired NETosis in these individuals. Whether the virus manipulates PAD4 expression, as an immune evasion mechanism, or whether individuals with lower PAD4 are more prone to SARS-CoV-2 infection, remains to be further investigated. Myocarditis is furthermore one of the hallmarks of COVID-19 [113] and roles for deimination/citrullination have previously been identified in a murine model of coxsackievirus B3-induced viral myocarditis [114]. As viral myocarditis was here identified as a KEGG pathway for deiminated proteins in cow sera and serum-EVs, to what extent PADs and deimination could be involved in viral induced myocarditis, including in relation to COVID-19, may be of considerable interest and remains to be further investigated. 

The complement and coagulation cascades were identified as a common KEGG immune pathway enriched in deiminated proteins in cow serum and serum-EVs. The complement system bridges innate and adaptive immunity, has roles in clearing apoptotic and necrotic cells and is in the front line for clearing invading pathogens [115,116,117,118,119]. Dysregulation of the complement system is furthermore associated with a range of pathologies [120]. In the current study a range of proteins from the complement cascade were identified as deiminated in cow serum and serum EVs, with complement components identified both in serum and serum-EVs including C1q, C3, C4A, C5a, C7, C8, C9 factor B, Factor H, C4-binding protein; while complement components found deiminated only in whole serum were C6, clusterin, and collectin-43 (CL43). C3 is the central component of the complement cascade and has furthermore been implicated in tissue remodeling during teleost ontogeny [121,122,123,124] as well as in regeneration [125]. A range of complement proteins have recently been identified by our group to be deiminated in a range of taxa throughout the phylogenetic tree including teleost and cartilaginous fish, camelids, cetaceans, pinnipeds, camelids and peglagic seabirds [16,17,91,92,93,94,95,96,98,99]. The bovine complement system [126] has been widely studied, including in immune defences against bovine anaplasmosis [127], in relation to heat-stress in dairy cows [128], susceptibility to bacterial infection relating to copper-deficiency [129]. Roles for modulation of complement regulation in relation to poxviral and pestiviral infections have been identified [130,131,132], including immune evasion by bovine herpesvirus 1 (BHV-1) [133], while and roles for antibody-dependent complement-mediated killing of mycoplasma have recently been revealed [134]. The finding of deiminated complement components highlight hitherto understudied roles for post-translational deimination in the known diversity of complement function throughout phylogeny [135,136,137,138,139]. Furthermore, immune evasion of bacteria (*Gingivalis*) by deimination of the host’s C5a has previously been identified [20] and indeed C5a was here identified as a deimination candidate in cow serum. Our findings suggest that protein deimination may play hitherto unidentified roles in the known unusual anti-pathogenic functions of cow serum, including via EV-transport, also contributing to complement function in homeostatic processes. 

Alpha-2-macroglobulin was found to be deiminated in cow serum and serum-EVs. It forms part of the innate immune system and clears active proteases from tissue fluids [140]. In cow plasma-EVs, alpha-2-macroglobulin has been identified [55], but the current study is the first report of deiminated bovine alpha-2-macroglobulin. Alpha-2-macroglobulin is related to the other thioester-containing proteins, complement proteins C3, C4 and C5, and is a phylogenetically conserved protein from arthropods to mammals [115,141,142]. The deimination of alpha-2-macroglobulin has recently been identified by our group in teleost fish, camelid, alligator and birds [15,92,93,99]. In the cow, alpha-2-macroglobulin has been widely studied and has amongst other binding affinities to TGF-beta and a range of cytokines [143] While alpha-2-macroglobulin is a known glycoprotein, less is known about deimination and such post-translational modification may facilitate protein moonlighting contribute both to its conserved immunological, as well as multifaceted functions in a range of taxa.

Serotransferrin was identified as a major deimination candidate in the current study both in cow serum and serum-EVs. It is an iron-binding protein with multifaceted functions in development and immunity [144,145]. Serotransferrin is found both in plasma and mucosal tissue and has important roles in anti-pathogenic responses across phylogeny, including in cattle, by withdrawing iron from pathogens including bacteria, fungi and viruses [146,147,148,149]. Furthermore, some viruses, including zoonitic ones, have been identified to utilize human transferrin receptor for viral entry and infection [150,151]. In chronic disease, for example lung diseases, the dysregulation of iron homeostasis can facilitate viral respiratory infections and secondary bacterial infections [152]. Deimination of serotransferrin has previously been identified by our group in teleost serum and mucosa [15,16]. Therefore, its deimination identified here in cow indicates that serotransferrin is deiminated in several taxa and this may be of considerable importance in its immune responses to a range of pathogens throughout phylogeny, as well as with respect to a range of human diseases and infections.

Intestinal immune network for IgA production was identified as deiminated for proteins common in serum and serum-EVs. The gut associated lymphoid system in the intestinal mucosa produces the highest level of antibody-secreting (IgA) plasma cells in the body [153,154]. It has been established that IgA cells play roles beyond antibody production, including having monocytic potential and antimicrobial properties via rapid secretion of cytokines as well as acting as regulators of inflammation [153]. IgA production in the gut mucosa plays major roles in maintaining the integrity of the mucosa and is therefore imperative for the host’s homeostasis and survival [154]. IgA is also produced in the BALT of the respiratory mucosal system [154] and is therefore important in defences against respiratory infections, including viral infections and this has also been identified in cattle bovine respiratory syncytial virus [155]. Deimination has been related to mucosal immunity in teleost fish, including in response to bacterial immune challenge and in EVs mediated mucosal immunity [15,16]. Roles for deimination in the regulation of intestinal immune networks for IgA production identified here may be of considerable interest and remain to be further investigated.

Various immunoglobulin (Ig) proteins and Ig superfamily members were identified here to be deiminated in cow serum and serum-EVs, confirming that Ig’s can be exported via EVs. Ig’s identified both in serum and serum-EVs were immunoglobulin lambda-like and immunoglobulin heavy chain constant mu. Ig’s identified in addition as deiminated in serum only, were the immunoglobulin J (joining) chain responsible for dimeric IgA and pentameric IgM and the polymeric immunoglobulin receptor (pIgR) that facilitates IgA’s translocation from the lamina propria through intestinal epithelia to the lumen. This is the first report of deiminated Ig’s in bovine serum and serum-EVs. Previous studies have identified that cow plasma EVs contain immunoglobulin J chain [55], but this did not come up as a deimination candidate in EVs, only in whole serum in the current study, and therefore may not be exported in EVs in the deiminated form. We have previously confirmed post-translational deimination if Ig’s in several other taxa, including shark, camelid, alligator and birds [17,92,93,99], as well as in teleost fish [15,16], and furthermore reported EV-mediated transport of Ig’s in shark and camelid [17,92]. Ig’s are key molecules in adaptive immunity and studied in diverse taxa. Post-translational deimination of Ig’s and roles in Ig functions remain to be further investigated but have been related to the IgG Fc region in bronchiectasis and RA [156]. Current understanding of Ig diversity throughout the phylogenetic tree is still incomplete [157,158,159,160,161,162]. Therefore, our identification of deimination of Ig’s in diverse taxa, including in bovine Ig’s in the current study, highlights a novel phylogenetically conserved concept of the diversification of Ig’s via this post-translational change. Our reported findings may furthermore shed some light on the unusual immune responses in bovine sera, relating to their immunoglobulins.

Serpins (serine proteases) were identified both in serum and serum-EVs, with both common candidates in serum and EVs as well as some specific serpin targets deiminated in serum or EVs. Serpins have multifaceted roles, ranging from protease inhibition to transport of hormones as well as regulation of chromatin organization [163]. Anti-viral roles for serine proteases have been identified in the airway, and serine proteases have been identified as drug targets for respiratory diseases, including virus infections [164,165]. Bovine serpins are cross-class inhibitors and shown to be strongly active against trypsin, as well as regulating caspases, therefore playing important roles in control of apoptosis in mammals [166]. In cow, serine proteases are for example involved in defences against *Babesia bovis*, a tick-borne and major apicomplexan pathogen in the cattle industry worldwide [167]. Serpins are identified in a range of taxa and for example serpin based peptides from alligator have been assessed as antimicrobials against multi-drug resistant pathogens [168]. Interestingly, serpins have been identified to be deiminated in alligator, and furthermore in humans, where deimination has been related to the modulation of protease activity and downstream effects on serpin-regulated pathways in rheumatoid arthritis [169]. As deimination of serpin pathways seems therefore a conserved phenomenon in a range of taxa, including in cattle as observed here, such post-translational regulation via deimination may contribute to its various functions, relating to immunity and anti-pathogenic defences, as well as also autoimmune diseases, and this remains to be further investigated. 

Fat digestion and absorption was identified as a KEGG pathway in both cow serum and serum-EVs alongside adiponectin as a specific target. The adipocytokine signalling pathway has key regulatory roles in metabolism and glucose regulation [170,171,172,173] and is linked to a range of pathologies, including type II diabetes and insulin resistance [174], to myopathies [175] and cancer [176]. Adiponectin has also been linked to longevity [177] and to regenerative functions [178]. In the cow, adiponectin is a known glycoprotein and has been assessed in bovine tissues as well as body fluids [179] and amongst other been identified as a pro-survival signal in ER stress in the mammary gland [180]. Adiponectin has recently been identified as a deimination candidate in several taxa with unusual metabolism, including camelids, the naked mole-rat, orca and alligator [92,93,96,98]. A range of apolipoproteins was furthermore identified to be deiminated in the current study in cow serum and serum-EVs. In cattle, apolipoprotein B-100, apolipoprotein A-I and apolipoprotein C-III have previously been identified as biomarkers for fatty liver and related diseases [181]. In other taxa, apolipoproteins have also been found to display antimicrobial activity against a range of pathogenic bacteria [182,183,184,185] as well as against viral (HIV) replication [186]. Various apolipoproteins have recently been identified as deimination protein candidates by our group in several taxa [15,16,92,93,99]. The deimination of apolipoproteins therefore needs further investigation in relation to their multifaceted phylogenic immune and metabolic functions.

Selenoprotein P (Sepp1) was identified to be deiminated in whole serum and serum-EVs. It is a plasma glycoprotein, mainly secreted from liver but also other tissues and contains most of the selenium in mammalian plasma [187,188,189]. In the cow roles for selenoproteins in mammary gland physiology and in milk formation have been identified [190] and roles for responses to oxidative stress in bovine arterial endothelial cells have also been suggested [191]. It has antioxidant properties [188] and serves in homeostasis and distribution of selenium [189]. Roles for selenoproteins in the regulation of the contraction and relaxation of bronchiolar smooth muscle have furthermore been identified [192]. Sepp1 is known to be glycosylated, and recently it has been identified as a deimination candidate in cetaceans, pinnipeds and alligators [93,96,97]. The contribution of deimination in the functional diversity and conserved functions of Sepp1 throughout phylogeny will remain to be further investigated.

KEGG pathways for ECM-receptor interactions were identified to be enriched in deiminated proteins in whole cow serum only. ECM-receptor interactions play both direct and indirect roles in controlling multifaceted cellular activities, such as cell differentiation, migration and proliferation as well as cell adhesion and apoptosis [193]. ECM-receptor interaction KEGG pathways have been identified in cancer [193] and enrichment for this pathway has been reported mesenchymal stem cell EVs [194]. Regulation of ECM-receptor interactions via post-translational deimination has recently been identified by our group via enrichment of deiminated proteins in KEGG pathways for ECM-receptor interactions in the fin whale, a long-lived cancer-resistant animal [96], in the alligator, an animal with unusual antibacterial and anti-viral responses [93], in the wandering albatross (*Diomedea exulans*), also an unusually long-lived bird for an avian species [99], as well as in aggressive brain cancer cells [100]. In the cow, ECM-receptor interactions are amongst other related to depot-specific adipogenesis [195] and found to be over-represented in specific cattle breeds [196]. ECM-receptor interaction pathway has furthermore been identified in Holstein cattle in relation to adaptive immune responses following viral vaccination [197] and in intra-mammary infection with *Streptococcus agalactiae* [198]. Therefore, deimination in this pathway may be of significant importance in anti-bacterial and anti-viral responses. Deimination in ECM-receptor interaction pathway is here described for the first time in cow and may play roles in the multifaceted functions of this pathway. 

KEGG pathways for calcium signalling pathway were here identified to be enriched in deiminated proteins in whole bovine serum only. Calcium is a key modulator in a range of immunological, metabolic and developmental functions [199,200,201,202]. Calcium signalling is linked to a wide range of physiological and pathological processes [203,204,205], is a key driver for post-translational deimination [206,207,208] and plays an important role in the regulation of EVs [11,48]. In cattle, calcium is linked to many immunological pathways including inflammatory diseases [209], virus replication [210], parasitic infection [211], wound healing [212], reproduction [213,214] and adrenal function [215]. Interestingly, calcium regulation has been shown to differ between Holstein and Jersey cows, including via serotonergic stimulation of the calcium pathway [216]. The identification in the current study of enrichment in deiminated proteins in the calcium signalling pathway in cow serum indicates a regulatory function in multiple key pathways of physiological and pathological processes via this post-translational modification. 

RAP1 (Ras-related protein 1) signalling pathway was identified as enriched in deiminated proteins in whole bovine serum. It belongs to a superfamily of small GTPases, with pleiotrophic regulatory functions in cellular processes, such as nuclear transport, cell cycle progression, vesicle trafficking, cell adhesion and cytoskeletal rearrangement [217]. Rap1 is an inflammatory regulator, is highly expressed in platelets and is a key molecule for platelet activation and adhesion in injury [218,219]. In mammals, Rap1 is a phylogenetically conserved telomere-interacting protein and promotes endothelial barrier function and angiogenesis [220,221], as well as being involved in cancer cell invasion and metastasis [222]. Rap1 also has roles in oxidative stress and metabolism, and besides cancer it is linked to metabolic and cardiac disorders, including cardiomyopathy [217] as well as Kabuki syndrome (characterised by congenital anomalities and developmental delay) [223]. Rap1 has also been linked to bone growth, including in bovine chondrocyte models [224]. Rap1 has been identified to undergo post-translational modifications by phosphorylation, geranylgeranylation or guanine nucleotide exchange factors [225], but deimination in this pathway has not been identified before, to our knowledge. To what extent deimination contributes to the multifaceted functions of Rap1 signalling in health and disease may therefore be of considerable interest.

Transcription misregulation in cancer and bladder cancer KEGG pathways were identified as enriched in deiminated proteins in bovine serum. Cows belong to a group of long-lived mammals that display cancer resistance and are considered a translationally valuable model for human cancers [2], including for breast cancer as they have been reported to be resistant to mammary cancer [226]. Cattle do generate bladder tumors and these have been studied in relation to various molecular mechanisms as well as in relation to viral infections, including papilloma and leukemia viruses, also in a comparative context to human cancer [103,227,228,229]. Bovine leukemia virus has furthermore been linked to telomerase upregulation and tumor induction in cattle [230]. PADs and deimination have been widely studied in a range of cancers, including via epigenetic regulation [9,10,11,100,231,232,233,234] but this is the first identification of post-translational regulation of such pathways via deimination in cattle.

HIF-1 (hypoxia inducible factor) signalling KEGG pathways were enriched in deiminated proteins in serum-EVs only. HIF-1 regulates oxygen homeostasis and is the key factor mediating the mammalian hypoxic response [235]. HIF-1 has roles in inflammation, cancer, angiogenesis and cardiovascular disease [236,237]. Furthermore, roles for HIF have been identified in bacterial infections [235] and a link between pathogenic agents and cancer has been identified, as viruses, bacteria and parasites may deregulate HIF-1 signalling associated cancer cells [238]. In cows, HIF-1 has for example been found to play roles in immune responses against *Theileria annulata*, a protozoan parasite of major economically importance for the cattle industry [239]. HIF-1 has also been identified to be important in angiogenesis and the maintenance of capillary structures for final follicle maturation in the cow ovary [240]. Furthermore, HIF-1 has recently been identified as a genomic marker differing between cattle breeds, including highland-adapted cattle [241]. Interestingly, enrichment in deiminated proteins in KEGG HIF-1 signalling pathways has previously been identified in EVs only, in some species with unusual immune and metabolic function including the naked mole-rat and deep-diving whales, both of which are animal models of hypoxia-tolerance and cancer-resistance [96,98]. Furthermore, deimination of HIF-1 regulation were recently identified in aggressive brain cancer (glioblastoma) cells [100]. To what extent deimination regulates HIF-1 signalling in relation to cancer, inflammation and infectious disease, including in cattle, remains to be further investigated.

KEGG pathways for biosynthesis of amino acids were identified as deiminated in serum-EVs only. This may be of considerable interest for comparative metabolic studies, particularly as amino acid assessment for mammalian metabolism and for research into ageing and disease has received some attention [242]. In dairy cows, these pathways are studies as they are of importance for understanding of optimization of diets and increased efficiency of microbial protein synthesis [243]. Enrichment for deiminated proteins in this KEGG pathway has previously been identified in alligator serum-EVs [93] as well as in cetacean sera [96] by our group, while mechanisms for deimination in this pathway remain to be elucidated.

The presence of deiminated histones H2A, H2B, H3 and H4 was identified in serum-EVs only, but not in whole bovine serum. Interestingly, a similar EV-mediated export of deiminated histones, as observed in cow serum-EVs in the current study, was recently identified in the naked mole-rat, an animal with unusual immunological and metabolic traits [98], as well as in alligator, also an animal with unusual antimicrobial, including anti-viral responses [93]. Deiminated histone H3 is commonly used as an indicator for neutrophil extracellular trap formation (NETosis) [31,244], which has been related to PAD4 and implicated in anti-viral responses in cattle respiratory syncytial virus disease [42], in response to parasitic infections in cattle [245,246,247] as well as in response to certain antibiotics [248]. Interestingly, in crocodilians, extracellular histones H2A and H4 have been identified to act as inhibitors of viral (HIV) infection in vitro [249], although roles for post-translational deimination were not assessed. Anti-microbial effects for histones have furthermore been observed in teleost fish mucosal immunity for H2A [250] as well as for deiminated histone H3 [15]. Histone H3 deimination has furthermore been linked to inflammatory and hypoxic responses during CNS regeneration in avian and murine models [25,26]. Histone deimination is also a known epigenetic mechanism in cancer [11,231,233]. The multifaceted functions of histones in immunity, including via post-translational regulation, such as deimination identified here, remains to be further investigated in cattle, including in relation to possible roles in anti-viral and other anti-pathogenic or disease related responses. 

The present study highlights novel aspects of protein moonlighting in immunity and metabolism of cattle, via post-translational deimination, including via EV-mediated transport. Our findings indicate differences in physiological and pathological pathways for deiminated proteins in serum-EVs, compared with whole serum, and may shed light on pathways underlying a number of pathological and anti-pathogenic (viral, bacterial, parasitic) pathways, with putative translatable value to human pathologies, zoonotic diseases and development of therapies for infections, including anti-viral therapies. While these data fall short of directing immediate clinical intervention for human disease, they do mandate further study of deimination in future work of broader scope and pre-clinical focus. For example, deimination will need further exploration as an additional diversification mechanism of ultralong CDR3 “cattlebodies” that have shown remarkable reach for broad viral neutralization. Also, importance for PADs in host-pathogen interactions, including in viral infections and in relation to COVID-19, may be of considerable importance and needs to be further investigated. 

## 4. Materials and Methods 

### 4.1. Serum Sampling from Cow

Blood was collected from the jugular vein of three healthy 5 months old Holstein breed steer and serum was prepared following clotting of blood for 2 h at room temperature (RT), by centrifugation for five minutes at 200× *g*. Sample collection was conducted under Texas A&M Institutional Animal Care and Use Protocol # 2015-078. Serum was aliquoted and kept at –80 °C until used. These samples were excess from a distinct study protocol and thus no animals needed to be purchased or euthanized for the study described here, consistent with the ethical principle of replacement, reduction and refinement.

### 4.2. Isolation of Extracellular Vesicles and Nanoparticle Tracking Analysis (NTA)

Serum-EVs were isolated from serum of individual animals (*n* = 3), using sequential centrifugation and ultracentrifugation in accordance to previously established protocols [17,46,98] and according to the recommendations of MISEV2018 (the minimal information for studies of extracellular vesicles 2018; [251]). For each individual EV preparation, 100 μL of bovine serum were diluted 1:5 in Dulbecco’s PBS (DPBS, ultrafiltered using a 0.22 μm filter, before use) and then centrifuged at 4000× *g* for 30 min at 4 °C, to ensure the removal of aggregates and apoptotic bodies. Thereafter the supernatants were collected and centrifuged further, using ultracentrifugation at 100,000× *g* for 1 h at 4 °C. The EV-enriched pellets were resuspended in 1 mL DPBS and ultracentrifuged again at 100,000× *g* for 1 h at 4 °C. The resulting washed EV pellets were then resuspended in 100 µL DPBS and frozen at –80 °C until further use. For EV size distribution profiles and EV quantification, NTA analysis was carried out using the NanoSight NS300 system (Malvern Panalytical, Malvern, UK), which analyses particle size based on Brownian motion. The EV samples were diluted 1/100 in DPBS (10 μL of EV preparation diluted in 990 μL of DPBS) and applied to the NanoSight using a syringe pump to ensure continuous flow of the sample. For each sample, five 60 second videos were recorded, keeping the number of particles per frame in-between 40 to 60. Replicate histograms were generated from the videos, using the NanoSight software 3.0 (Malvern), representing mean and confidence intervals of the 5 recordings for each sample. 

### 4.3. Transmission Electron Microscopy (TEM)

A pool of EVs, isolated from serum of the three individual animals as described above, was used for morphological analysis using TEM. Following isolation, the EVs were resuspended in 100 mM sodium cacodylate buffer (pH 7.4) and a drop (~3–5 μL) of the suspension was placed onto a grid with previously glow discharged carbon support film. After the suspension had partly dried, the EVs were fixed by placing the grid onto a drop of a fixative solution (2.5% glutaraldehyde in 100 mM sodium cacodylate buffer (pH 7.0)) for 1 min at room temperature and washed afterwards by touching the grid to the surface of three drops of distilled water. Excess water was removed by touching the grid to a filter paper. Next, the EVs were stained with 2% aqueous Uranyl Acetate (Sigma-Aldrich) for 1 min, the excess stain was removed by touching the grid edge to a filter paper and the grid was let to dry. Imaging of EVs was performed using a JEOL JEM 1400 transmission electron microscope (JEOL, Tokyo, Japan) operated at 80 kV at a magnification of 30,000× to 60,000×. Digital images were recorded using an AMT XR60 CCD camera (Deben UK Limited, Bury Saint Edmunds, UK).

### 4.4. Isolation of Deiminated Proteins in Serum and EVs Using F95-enrichment

Immunoprecipitation and isolation of deiminated proteins in serum and serum-derived EVs was carried out using the Catch and Release^®^v2.0 immunoprecipitation kit (Merck, UK) in conjunction with the F95 pan-deimination antibody (MABN328, Merck), which has been developed against a deca-citrullinated peptide and specifically detects proteins modified by citrullination [252]. Bovine serum pools of the three individual animals (3 × 25 μL) were used for F95-enrichment from whole serum, while for EVs, total protein was first extracted from the pool of EVs derived from 3 animals (EV pellets derived from 100 μL serum per animal), using RIPA+ buffer (Sigma, UK). Following application of RIPA+ buffer, the EVs were incubated on ice for 2 h followed by centrifugation at 16,000× *g* for 30 min to collect the protein containing supernatant. Thereafter, immunoprecipitation (F95-enrichment) was carried out overnight on a rotating platform at 4 °C. The F95 bound proteins were eluted using denaturing elution buffer (Merck), according to the manufacturer’s instructions (Merck) and diluted 1:1 in Laemmli sample buffer. The F95-enriched eluates from whole serum and serum -EVs were then analyzed by SDS-PAGE, followed by silver staining, Western blotting or LC-MS/MS.

### 4.5. Western Blotting Analysis

Bovine serum and serum-EVs were diluted 1:1 in denaturing 2× Laemmli sample buffer (containing 5% beta-mercaptoethanol, BioRad, Kidlington, UK) and boiled for 5 min at 100 °C. Proteins were separated by SDS-PAGE using 4%-20% gradient TGX gels (BioRad UK). Western blotting was carried out using the Trans-Blot^®^ SD semi-dry transfer cell (BioRad, UK); even transfer was assessed by staining the membranes with PonceauS (Sigma, UK). Blocking was performed for 1 h at room temperature using 5% bovine serum albumin (BSA; Sigma, UK), in Tris buffered saline (TBS) containing 0.1% Tween20 (BioRad, UK; TBS-T). Following blocking the membranes were incubated overnight at 4 °C on a shaking platform with the primary antibodies, which were diluted in TBS-T. For detection of deiminated/citrullinated proteins, the F95 pan-deimination antibody was used (MABN328, Merck, 1/1000). For detection of PAD proteins in bovine serum, the following anti-human PAD antibodies were used: anti-PAD2 (ab50257, Abcam, Cambridge, 1/1000); anti-PAD3 (ab50246, Abcam, 1/1000) and anti-PAD4 (ab50247, Abcam, 1/1000), and have previously been shown to cross-react with PAD homologues in a range of taxa [15,16,17,25,26,92,93,96,97,98,99]. EV isolates were blotted against two EV-specific markers: CD63 (ab216130, 1/1000) and Flotillin-1 (Flot-1, ab41927, 1/2000), for characterisation of EVs. After primary antibody incubation the membranes were washed for 3 × 10 min in TBS-T at RT and incubated for 1 h, at RT with HRP-conjugated secondary antibodies (anti-rabbit IgG (BioRad) or anti-mouse IgM (BioRad) respectively, diluted 1/3000 in TBS-T). The membranes were then washed in TBS-T for 5 × 10 min and positive proteins bands were visualized digitally, using enhanced chemiluminescence (ECL, GE Healthcare, Amersham Pl, Little Chalfont, UK) and the UVP BioDoc-ITTM System (Thermo Fischer Scientific, Hemel Hempstead, UK). 

### 4.6. Silver Staining

F95-enriched protein eluates from bovine serum and serum-EVs were silver stained following SDS-PAGE (using 4–20% gradient TGX gels, BioRad, UK) under reducing conditions, using the BioRad Silver Stain Plus Kit (1610449, BioRad, UK), according to the manufacturer’s instructions (BioRad).

### 4.7. Liquid Chromatography with Tandem Mass Spectrometry (LC-MS/MS) Analysis of Deiminated Protein Candidates

F95-enriched eluates from bovine serum and serum-EVs respectively, were analyzed by liquid chromatography with tandem mass spectrometry (LC-MS/MS), according to previously described methods [93,99]. For LC-MS/MS analysis, the F95-enriched eluates were run 0.5 cm into a 12% TGX gel (BioRad, UK), the band cut out, trypsin digested and subjected to proteomic analysis on a Dionex Ultimate 3000 RSLC nanoUPLC (Thermo Fisher Scientific Inc, Waltham, MA, USA.) system and a QExactive Orbitrap mass spectrometer (Thermo Fisher Scientific Inc, Waltham, MA, USA) according to previously described methods [93,99]. In brief, peptides were separated by reverse-phase chromatography and a Thermo Scientific reverse-phase nano Easy-spray column (Thermo Scientific PepMap C18, 2 µm particle size, 100A pore size, 75 µm i.d. × 50 cm length). First, a pre-column (Thermo Scientific PepMap 100 C18, 5 µm particle size, 100A pore size, 300 µm i.d. × 5 mm length) was used for loading the peptides from the Ultimate 3000 autosampler, in the presence of 0.1% formic acid for 3 min (flow rate 10 µL/min). Therafter, peptides were eluted from the pre-column onto the analytical column (solvent A = water + 0.1% formic acid; solvent B = 80% acetonitrile, 20% water + 0.1% formic acid). A linear gradient of 2–40% B was applied for 30 min. An easy-Spray source (Thermo Fischer Scientific Inc., Waltham, MA, USA) as used to spray the LC eluant into the mass spectrometer. The *m*/*z* values of the eluting ions were measured using an Orbitrap mass analyzer. The resolution was set at 70,000 and scanning was performed at *m*/*z* 380–1500. Fragment ions were automatically isolated and generated based on data dependent scans (top 20) in the HCD collision cell by higher energy collisional dissociation (HCD, NCE:25%). The resulting fragment ions were measured using the Orbitrap analyzer, set at a resolution of 17500. Ions that were singly charged, or with unassigned charge states, were excluded from selection for MS/MS. Furthermore, a dynamic exclusion window of 20 seconds was applied. The Protein Discoverer (version 2.1., Thermo Scientific) was used to process the data post-run and the MS/MS data was thereafter converted to mgf files. For identification of deiminated protein hits, the files were then submitted to the Mascot search algorithm (Matrix Science, London, UK) and searched against the UniProt *Bos taurus*_ 20170607 database (24148 sequences; 40594 residues) and *Bos_primigenius* primigenius_20191209 database (54 sequences; 14783 residues), as well as a common contaminant sequences (123 sequences; 40594 residues). Fragment and peptide mass tolerances and were set at 0.1 Da and 20 ppm, respectively. The peptide cut-off score was set at 31 and significance at *p* < 0.05 (carried out by Cambridge Proteomics, Cambridge, UK). 

### 4.8. Protein–Protein Interaction Network Analysis

For the identification and prediction of putative protein–protein interaction networks for deiminated proteins identified in bovine serum and serum-EVs, STRING analysis (Search Tool for the Retrieval of Interacting Genes/Proteins; https://string-db.org/) was used. Protein networks were built based on the protein IDs and using the function of “search multiple proteins” in STRING, choosing “*Bos taurus*” for the species database. For analysis, “medium conficence” and “basic settings” were selected and the colour lines connecting nodes indicated the following evidence-based interactions for the network edges: “known interactions”, which are based on curated databases or experimentally determined interactions; “predicted interactions”, which are based on protein homology, co-expression, gene fusion, gene neighborhood, gene co-occurrence, or established by text mining.

### 4.9. Phylogenetic Comparison of Bos PADs with Human PADs

A neighbor-joining phylogeny tree was constructed using Clustal Omega (https://www.ebi.ac.uk/Tools/msa/clustalo/) based on sequence alignment of amino acid sequences for bovine PADs 1-6 (PAD1, NP_001094742.1; PAD2, NP_001098922.1; PAD3, XP_010800991.1; PAD4, NP_001179102.1 and PAD6, XP_002685843.1) compared with human PADs 1-6 (PAD1, NP_037490.2; PAD2, NP_031391.2; PAD3, NP_057317.2; PAD4, NP_036519.2 and PAD6, NP_997304.3).

### 4.10. Statistical Analysis

The histograms and graphs were prepared using the Nanosight 3.0 software (Malvern Panalytical) and GraphPad Prism version 7 (GraphPad Software, San Diego, CA, USA). NTA curves represent mean and standard error of mean (SEM), indicated by confidence intervals. STRING analysis (https://string-db.org/) was used for prediction of protein–protein interaction networks. Significance was considered as *p* ≤ 0.05.

## 5. Conclusions

In the current study we report for the first time deimination profiles of serum and serum-EVs of *Bos taurus*. Post-translational deimination of key immune, metabolic and nuclear proteins was identified and these related to KEGG pathways for a number of immunological pathways with relevance to viral, bacterial and parasitic infections, to chronic and autoimmune diseases, as well as a range of metabolic pathways. Our findings highlight novel aspects of protein moonlighting in immunity and metabolism of cattle, via post-translational deimination, including via EV-mediated transport.

While these data fall short of directing immediate clinical intervention for human disease, they do mandate further study of deimination as an additional diversification mechanism of ultralong CDR3 “cattlebodies” that have shown remarkable reach for broad viral neutralization. This study limitation of this comparative biochemical basic science must be addressed in future work of broader scope and pre-clinical focus. Furthermore, transcriptomics data revealing downregulation in PAD4 mRNA levels in SARS-CoV-2 infected patient’ lung biopsies, compared to healthy controls (https://www.ncbi.nlm.nih.gov/bioproject/PRJNA615032), indicates importance for PADs in host-virus interactions in COVID-19 and will need to be further explored.

EVs research in comparative immunology and veterinary sciences is a rapidly growing field and while EVs have been previously assessed in cattle, this study is the first to characterise deiminated protein signatures in bovine serum and serum-EVs. The identification of post-translational deimination and EV-mediated communication in bovine immunity and physiology, revealed here, contributes to current understanding of protein moonlighting functions and EV-mediated communication in cattle, providing novel insight into their unusual immune systems and physiological traits. Our findings may shed light on pathways underlying a number of pathological and anti-pathogenic, including anti-viral, pathways, with putative translatable value to human pathologies, zoonotic diseases and development of therapies for infections, including anti-viral therapies.

## Figures and Tables

**Table 1:** Deiminated proteins in bovine serum (*Bos taurus*), as identified by F95-enrichment and LC-MS/MS analysis. Deiminated proteins from serum were isolated by immunoprecipitation using the pan-deimination F95 antibody. The resulting F95-enriched eluate was then analysed by LC-MS/MS and peak list files submitted to Mascot. *Bos taurus* species-specific peptide sequence hits are listed, showing number of sequences for protein hits and total score. Blue highlighted rows indicate protein hits identified in whole sera only. Uncharacterised proteins based on LC-MS/MS search were further confirmed in STRING, based on the protein symbol, and protein identification from STRING, where retrieved, is shown in brackets ([]). For further full LC-MS/MS analysis of all protein hits see Supplementary Table S1.

Protein Name	Symbol	Sequences	Total Score (*p* < 0.05) ^1^
Serotransferrin	G3X6N3	76	5712
Serotransferrin	Q29443	76	5711
Complement factor H	Q28085	77	5123
Alpha-2-macroglobulin	Q7SIH1	77	5079
Serum albumin	A0A140T897	59	3944
Uncharacterised protein [complement factor H]	F1MC45	54	3745
Complement C3	Q2UVX4	68	3483
Uncharacterised protein [ceruloplasmin]	F1N076	44	2712
Embryo-specific fibronectin 1	B8Y9S9	37	2263
Hemopexin	Q3SZV7	30	2019
Uncharacterised protein [complement C4A]	E1BH06	34	1997
C4b-binding protein alpha chain	Q28065	33	1841
Kininogen-1	A0A140T8C8	23	1505
Kininogen-1	P01044	22	1449
Histidine-rich glycoprotein	F1MKS5	26	1432
Uncharacterised protein (uncharacterised)	F1MJK3	26	1291
Kininogen-2	P01045	19	1271
Uncharacterised protein (uncharacterised)	G3X7F3	16	1259
Uncharacterised protein (uncharacterised)	G3N0V0	19	1186
Apolipoprotein A-I preproprotein	V6F9A2	19	1122
Uncharacterised protein (uncharacterised)	F1MVK1	17	953
SERPIND1 protein	A6QPP2	16	865
Uncharacterised protein (uncharacterised)	F1MZ96	13	837
Uncharacterised protein [*Bos taurus* immunoglobulin lambda-like polypeptide 1 (IGLL1)]	F1MLW7	10	815
Uncharacterised protein [*Bos taurus* immunoglobulin lambda-like polypeptide 1 (IGLL1)]	F1MCF8	10	796
Uncharacterised protein [Immunoglobulin heavy constant mu]	G5E5T5	12	784
Uncharacterised protein [Immunoglobulin heavy constant mu]	G5E513	10	732
Uncharacterised protein (uncharacterised)	G3N0S9	12	648
Histidine-rich glycoprotein	P33433	12	623
Alpha-2-antiplasmin	P28800	11	531
Uncharacterised protein (uncharacterised)	F1MLW8	5	434
Hemoglobin subunit beta	P02070	6	422
Vitamin D-binding protein	F1N5M2	9	420
Alpha-1-antiproteinase	P34955	8	418
Adiponectin	Q3Y5Z3	6	411
Uncharacterised protein (uncharacterised)	F1MW79	7	403
ECM1 protein	A5PJT7	8	395
Complement C1q subcomponent subunit B	Q2KIV9	8	390
Plasma kallikrein	Q2KJ63	8	385
Inter-alpha-trypsin inhibitor heavy chain H4	F1MMD7	6	367
C1QC protein	Q1RMH5	5	347
Uncharacterised protein [Serpin A3-5; Serine protease inhibitor]	G8JKW7	6	338
Apolipoprotein A-IV	F1N3Q7	7	338
Inter-alpha-trypsin inhibitor heavy chain H2	F1MNW4	6	338
Selenoprotein P	P49907	6	323
Plasminogen	E1B726	6	302
Complement C1q subcomponent subunit A	Q5E9E3	5	300
Uncharacterised protein [*Bos taurus* insulin-like growth factor binding protein, acid labile subunit (IGFALS)]	F1MJZ4	6	277
Thrombospondin-1	F1N3A1	7	273
Serpin A3-3	G3N1U4	5	271
Protein HP-20 homolog	Q2KIT0	5	269
Uncharacterised protein (uncharacterised)	G5E604	4	234
Inter-alpha-trypsin inhibitor heavy chain H1	Q0VCM5	4	234
Uncharacterised protein (uncharacterised)	E1B805	5	232
Uncharacterised protein (uncharacterised)	G3MXD9	3	209
Uncharacterised protein [*Bos taurus* immunoglobulin lambda-like polypeptide 1 (IGLL1)]	G3N2D7	3	204
Keratin, type II cytoskeletal 7	Q29S21	4	199
Keratin, type I cytoskeletal 14	F1MC11	4	199
Uncharacterised protein [Heparan sulfate proteoglycan 2]	F1MER7	4	195
Alpha-2-HS-glycoprotein	cRAPR1|FETUA_BOVIN	4	190
Uncharacterised protein (uncharacterised)	G3N1H5	2	187
Serpin A3-8	A0A0A0MP89	3	184
Complement factor B	P81187	4	183
Protein AMBP	F1MMK9	5	181
Serpin A3-2	A2I7M9	4	180
Antithrombin-III	F1MSZ6	5	176
Paraoxonase 1	Q2KIW1	4	174
Gelsolin	F1MJH1	3	171
Uncharacterised protein (uncharacterised)	G3N3Q3	2	168
Complement C5a anaphylatoxin	F1MY85	3	166
Uncharacterised protein (uncharacterised)	G5E5H2	3	162
Serpin A3-7	A0A0A0MP92	4	160
C1QTNF3 protein	A7MB82	3	160
Uncharacterised protein [*Bos taurus* serotransferrin-like]	E1BI82	4	157
Uncharacterised protein [Apolipoprotein B]	E1BNR0	4	150
Primary amine oxidase, liver isozyme	Q29437	3	137
Coagulation factor XI	F1MUT4	2	127
Hepatocyte growth factor-like protein	E1BDW7	2	121
Fibrinogen beta chain	F1MAV0	3	108
Immunoglobulin J chain	Q3SYR8	2	107
Uncharacterised protein (uncharacterised)	G3MXB5	2	100
Uncharacterised protein [CD5 molecule-like]	F1N514	2	94
Uncharacterised protein (uncharacterised)	G3MXG6	2	94
Complement component C9	Q3MHN2	2	87
Complement component C6	F1MM86	3	86
Uncharacterised protein (uncharacterised)	G5E5V1	2	86
Hemoglobin subunit alpha	P01966	2	85
Uncharacterised protein (uncharacterised)	G3N028	2	75
Fibrinogen alpha chain	A5PJE3	1	74
Vitronectin	Q3ZBS7	2	73
Alpha-1B-glycoprotein	Q2KJF1	1	66
Coagulation factor XII	F1MTT3	1	65
Uncharacterised protein (uncharacterised)	F1MSF0	2	63
Prothrombin	P00735	1	61
Uncharacterised protein [Nidogen 1]	F1MWN3	1	57
Protein HP-25 homolog 2	Q2KIU3	1	57
Clusterin	F1MWI1	1	55
Contactin-1	F1MVI0	1	54
Polymeric immunoglobulin receptor	F1MR22	2	51
Uncharacterised protein [Sperm associated antigen 9]	F1MZ69	2	51
Uncharacterised protein [Ankyrin repeat domain 11]	E1BAT5	2	51
Beta-2-glycoprotein 1	A0A140T843	1	50
Carboxypeptidase N catalytic chain	Q2KJ83	1	49
Uncharacterised protein [complement component 8, alpha polypeptide (C8A)]	F1MX87	1	48
Fibrinogen gamma-B chain	F1MGU7	1	48
Uncharacterised protein [ADAM metallopeptidase with thrombospondin type 1 motif, 13]	F1MVP0	1	47
Uncharacterised protein [Talin 1]	F1MDH3	1	46
Complement C1s subcomponent	Q0VCX1	1	46
Uncharacterised protein [CL43—Collectin-43]	F1MFY6	1	45
Complement component C7	F1N045	1	44
CLEC11A protein	A5D7L1	1	43
Uncharacterised protein [KCTD12—Potassium channel tetramerization domain containing 12]	G3N3D4	1	43
Uncharacterised protein [BRIP1—BRCA1 interacting protein C-terminal helicase 1]	E1BNG9	1	39
Cadherin-1	F1N619	1	39
Ubiquitin carboxyl-terminal hydrolase isozyme L5	Q9XSJ0	1	38
CPN2 protein	A6QP30	1	37
Acyl-coenzyme A thioesterase THEM4	A1A4L1	1	37
Uncharacterised protein [SLAMF9—SLAM family member 9]	E1BNF9	1	36
Uncharacterised protein [MED12; Mediator complex subunit 12]	F1MZ95	1	35
Transthyretin	O46375	1	33
Voltage-dependent L-type calcium channel subunit beta-3	Q9MZL3	1	33

^1^ Ions score is −10*Log(P), where *P* is the probability that the observed match is a random event. Individual ions scores > 31 indicated identity or extensive homology (*p* < 0.05). Protein scores were derived from ions scores as a non-probabilistic basis for ranking protein hits.

**Table 2:** Deiminated proteins in serum-EVs of cow (*Bos taurus*) as identified by F95-enrichment. Deiminated proteins from serum-EVs were isolated by immunoprecipitation using the pan-deimination F95 antibody. The resulting F95-enriched eluate was then analysed by LC-MS/MS and peak list files submitted to Mascot. *Bos taurus* species-specific peptide sequence hits are listed, showing number of sequences for protein hits and total score. Rows highlighted in pink indicate protein hits identified in serum-EVs only. Uncharacterised proteins based on LC-MS/MS search were further confirmed in STRING, based on the protein symbol, and protein identification from STRING, where retrieved, is shown in brackets ([]).For further full LC-MS/MS analysis of all protein hits see Supplementary Table S2.

Protein Name	Symbol	Sequences	Total Score (*p* < 0.05) ^1^
Serotransferrin	G3X6N3	75	4958
Serotransferrin	Q29443	74	4941
Complement factor H	Q28085	85	4700
Alpha-2-macroglobulin	Q7SIH1	77	4633
Serum albumin	A0A140T897	76	4604
Complement C3	Q2UVX4	83	4258
Uncharacterised protein (CFH—Complement factor H)	F1MC45	61	3469
Fibronectin	G5E5A9	56	2492
Uncharacterised protein (Ceruloplasmin)	F1N076	37	1994
Uncharacterised protein (uncharacterised)	F1MJK3	38	1870
Uncharacterised protein (C4A—Complement C4)	E1BH06	35	1840
C4b-binding protein alpha chain	Q28065	36	1761
Keratin, type II cytoskeletal 5	M0QVZ6	35	1750
Hemopexin	Q3SZV7	30	1594
Keratin, type I cytoskeletal 14	F1MC11	29	1592
Uncharacterised protein (uncharacterised)	G3N0V0	25	1542
Histidine-rich glycoprotein	F1MKS5	26	1407
Uncharacterised protein (keratin 33A (KRT33A))	F1MXG6	22	1311
KRT33A protein	A5PJJ1	22	1306
Keratin 31	Q148I8	22	1291
Uncharacterised protein (uncharacterised)	G3X7F3	20	1288
Keratin, type II cuticular Hb1	Q148H4	25	1225
Uncharacterised protein (keratin 34 (KRT34))	F1MSA6	21	1209
Kininogen-1	A0A140T8C8	21	1188
Kininogen-1	P01044	21	1164
Uncharacterised protein (Desmoplakin)	E1BKT9	27	1156
Uncharacterised protein (keratin 86 (KRT86))	E1B898	22	1104
Apolipoprotein A-I preproprotein	V6F9A2	22	1101
Uncharacterised protein (uncharacterised)	F1MVK1	20	1066
Uncharacterised protein (KRT6A—Keratin, type II cytoskeletal 59 kDa, component IV)	M0QVY0	21	1049
Kininogen-2	P01045	19	1042
Uncharacterised protein (KRT3—Keratin, type II cytoskeletal 68 kDa, component IB)	G3MXL3	21	1034
Junction plakoglobin	Q8SPJ1	21	987
Keratin, type II cytoskeletal	Q08D91	19	971
Uncharacterised protein (Immunoglobulin heavy constant mu)	G5E5T5	16	938
Keratin, type I cytoskeletal 17	A0A140T867	17	890
Uncharacterised protein (IGLL1—immunoglobulin lambda-like polypeptide 1)	F1MCF8	12	886
Uncharacterised protein (uncharacterised)	F1MH40	13	872
Uncharacterised protein (uncharacterised)	F1MZ96	14	863
Keratin 10 (Epidermolytic hyperkeratosis; keratosis palmaris et plantaris)	A6QNZ7	15	854
Uncharacterised protein (uncharacterised)	F1MLW7	10	849
Complement C4	P01030	16	826
Uncharacterised protein (Immunoglobulin heavy constant mu)	G5E513	15	810
KRT15 protein	Q17QL7	13	734
Uncharacterised protein (uncharacterised)	G3N0S9	14	631
Keratin, type I cytoskeletal 19	P08728	12	625
KRT4 protein	A4IFP2	12	623
SERPIND1 protein	A6QPP2	12	606
Uncharacterised protein (uncharacterised)	F1MLW8	7	588
Histidine-rich glycoprotein	P33433	9	566
Inter-alpha-trypsin inhibitor heavy chain H2	F1MNW4	12	554
Inter-alpha-trypsin inhibitor heavy chain H4	F1MMD7	10	521
Vitamin D-binding protein	F1N5M2	12	517
Antithrombin-III	F1MSZ6	11	492
Plasminogen	P06868	14	485
Apolipoprotein A-IV	Q32PJ2	11	475
Serpin A3-3	G3N1U4	9	442
Complement factor B	P81187	9	429
C1QC protein	Q1RMH5	8	428
Complement C1q subcomponent subunit B	Q2KIV9	7	413
Uncharacterised protein (serotransferrin-like)	E1BI82	8	411
Uncharacterised protein (KRT16—Keratin 16)	G3X7W8	9	407
Complement C1q subcomponent subunit A	Q5E9E3	7	401
Serpin A3-4	A2I7N0	9	383
Alpha-1B-glycoprotein	Q2KJF1	7	381
Uncharacterised protein (uncharacterised)	G3N3Q3	5	367
Uncharacterised protein (Serpin A3-5)	G8JKW7	7	366
Keratin, type II cytoskeletal 78	A6QNX5	7	356
Primary amine oxidase, liver isozyme	Q29437	8	352
Serpin A3-2	A2I7M9	7	333
Uncharacterised protein (uncharacterised)	F1MW79	6	330
Uncharacterised protein (uncharacterised)	G3N1H5	5	325
Uncharacterised protein (KRT77—Keratin 77)	G3MYU2	6	310
Uncharacterised protein (uncharacterised)	E1B805	7	309
Uncharacterised protein (uncharacterised)	G3MXG6	4	305
Uncharacterised protein (uncharacterised)	G3MXD9	5	300
Uncharacterised protein (uncharacterised)	G3N2P6	5	292
Uncharacterised protein (insulin-like growth factor binding protein, acid labile subunit (IGFALS))	F1MJZ4	5	286
Adiponectin	Q3Y5Z3	5	282
Alpha-2-antiplasmin	P28800	7	271
Protein AMBP	F1MMK9	6	268
Vitronectin	Q3ZBS7	5	264
Uncharacterised protein (uncharacterised)	G5E604	5	254
Desmoglein-1	F1MIW8	7	252
Fibrinogen gamma-B chain	F1MGU7	5	252
Uncharacterised protein (uncharacterised)	G3MY71	5	251
Annexin A2	P04272	5	244
Fibrinogen alpha chain	A5PJE3	5	243
Uncharacterised protein (uncharacterised)	G3MWT1	3	241
Glyceraldehyde-3-phosphate dehydrogenase	P10096	6	238
Serpin A3-7	A0A0A0MP92	5	218
Plakophilin-1	Q28161	4	216
Inter-alpha-trypsin inhibitor heavy chain H1	Q0VCM5	5	213
Hemoglobin subunit beta	P02070	4	212
Alpha-1-antiproteinase	P34955	4	209
Keratin, type II	A0JND2	4	198
Actin, cytoplasmic 1	F1MRD0	5	197
Keratin, type I cytoskeletal 28	Q148H6	4	194
Thrombospondin-1	F1N3A1	6	192
Keratin, type I cytoskeletal 20	F1MPK1	4	191
ECM1 protein	A5PJT7	5	188
Uncharacterised protein (*Bos taurus* immunoglobulin lambda-like polypeptide 1 (IGLL1))	G3N2D7	3	186
Selenoprotein P	P49907	5	184
Uncharacterised protein (uncharacterised)	A0A0A0MPA0	4	176
Gelsolin	F1MJH1	4	175
Lactotransferrin	P24627	4	169
Fibrinogen beta chain	F1MAV0	5	162
Uncharacterised protein (uncharacterised)	G5E5H2	3	156
Protein HP-20 homolog	Q2KIT0	3	151
Uncharacterised protein (TGM1—Transglutaminase 1)	F1MBB7	2	143
Actin, gamma-enteric smooth muscle	F1MKC4	4	143
Uncharacterised protein (CD5L—CD5 molecule-like)	F1N514	4	131
Complement component C7	F1N045	4	128
Uncharacterised protein (APOB—Apolipoprotein B)	E1BNR0	4	119
Arginase-1	Q2KJ64	3	119
Alpha-S1-casein	CASA1_BOVIN	2	115
Uncharacterised protein (uncharacterised)	G3N028	2	114
Alpha-2-HS-glycoprotein	cRAPR1|FETUA_BOVIN	2	106
Paraoxonase 1	Q2KIW1	3	106
Beta-2-glycoprotein 1	A0A140T843	2	97
Uncharacterised protein (uncharacterised)	G5E5V1	2	89
Uncharacterised protein (Histone H2B family)	E1B7N8	2	88
Complement component 1, r subcomponent	A5D9E9	3	83
Histone H2A	A0A0A0MP90	2	80
Uncharacterised protein (SLK-STE20-like kinase)	G3X696	3	80
Prothrombin	P00735	2	79
Peroxiredoxin-2	Q9BGI3	3	79
Uncharacterised protein (uncharacterised)	G3MZE0	1	69
Uncharacterised protein (complement component 8, alpha polypeptide (C8A))	F1MX87	1	69
Uncharacterised protein (C1orf68—Chromosome 1 open reading frame 68)	G3N3D3	2	68
Complement C5a anaphylatoxin	F1MY85	2	63
Uncharacterised protein (complement factor I (CFI))	F1N4M7	1	61
Uncharacterised protein (armadillo repeat gene deleted in velocardiofacial syndrome (ARVCF))	E1BPV1	2	59
Uncharacterised protein (NEK10-Uncharacterised protein; NIMA-related kinase 10)	E1BHZ1	2	58
Keratin associated protein 13-1	A1A4M9	2	57
Uncharacterised protein (uncharacterised)	F1MSF0	2	57
Histone H4	E1B9M9	1	56
Gamma-glutamylcyclotransferase	Q32LE4	1	55
Uncharacterised protein (JAKMIP2—Janus kinase and microtubule interacting protein 2)	G5E551	2	54
TOX high mobility group box family member 4	Q0P5K4	1	53
Uncharacterised protein (TBC1D32—TBC1 domain family, member 32)	F1N7V1	2	51
Proteasome subunit alpha type-6	Q2YDE4	1	50
Uncharacterised protein (KIAA1009 ortholog)	F1MZ01	2	50
Uncharacterised protein (MYO5B—Myosin VB)	F1MMQ6	2	50
Uncharacterised protein (Phospholipid phosphatase related 4)	F1MJ26	2	49
Uncharacterised protein (MGA—MGA, MAX dimerization protein)	E1BKB7	2	49
Uncharacterised protein (GOLGB1—Golgin B1)	E1BKZ5	2	47
Histone H3	E1BGN3	1	46
Uncharacterised protein (CROCC—Ciliary rootlet coiled-coil, rootletin)	E1BBS9	3	46
Protein FAM149B1	A0JNF3	2	45
Alpha-enolase	F1MB08	2	45
Uncharacterised protein (UBR4—Ubiquitin protein ligase E3 component n-recognin 4)	E1BHT5	3	45
Complement component C9	Q3MHN2	1	44
Uncharacterised protein (DOCK8—*Bos taurus* dedicator of cytokinesis 8 (DOCK8))	E1BNA6	2	44
Uncharacterised protein (KPRP—Keratinocyte proline-rich protein)	E1BLN6	1	44
Uncharacterised protein (CCDC14—Coiled-coil domain containing 14)	F1MS02	2	43
Uncharacterised protein (Strawberry notch homolog 1(Drosophila))	E1BMP8	3	42
DNA-directed RNA polymerase subunit beta	A5PJW8	1	42
Uncharacterised protein (CRYGN-Crystallin, gamma N)	E1BDQ1	1	42
Flotillin-1	Q08DN8	1	41
Uncharacterised protein (SMG6—Uncharacterised protein; *Bos taurus* smg-6 homolog, nonsense mediated mRNA decay factor (*C. elegans*) (SMG6))	E1BFK4	2	41
C1QTNF3 protein	A7MB82	1	41
Uncharacterised protein (Protocadherin gamma subfamily B, 1)	F1MCA2	1	40
Uncharacterised protein (Death-associated protein kinase 1)	F1MRL0	1	39
Acyl-coenzyme A thioesterase THEM4	A1A4L1	1	37
Uncharacterised protein (Centrosomal protein 104kDa)	E1BND2	1	37
C8G protein	A8YXZ2	1	36
Uncharacterised protein (SERPINB12—Serpin family B member 12)	E1BDF5	1	36
Uncharacterised protein (SLAMF9 SLAM family member 9)	E1BNF9	1	36
Complement C1s subcomponent	Q0VCX1	1	36
Uncharacterised protein (methyltransferase like 3 (METTL3))	F1MX80	1	35
Uncharacterised protein (SPAG9—Sperm associated antigen 9)	F1MZ69	1	35
Adenosylhomocysteinase	Q3MHL4	1	35
Uncharacterised protein (HIVEP3—Human immunodeficiency virus type I enhancer binding protein 3)	F1MBK6	1	34
Coagulation factor V	F1N0I3	1	33
Uncharacterised protein (VPREB1—Uncharacterised protein)	F1N160	1	33
Uncharacterised protein (SERPING1)	E1BMJ0	1	33
PPARD protein	A4IFL4		32
Uncharacterised protein	F1MZ93	1	32
Uncharacterised protein (NCBP1—Nuclear cap binding protein subunit 1)	E1BMM0	1	32
Transthyretin	O46375	1	32
Uncharacterised protein (Cryptochrome-1)	F1MXB2	1	32
Tubulin alpha chain	F1MNF8	1	31

**^1^** Ions score is −10*Log(P), where P is the probability that the observed match is a random event. Individual ions scores > 31 indicated identity or extensive homology (*p* < 0.05). Protein scores were derived from ions scores as a non-probabilistic basis for ranking protein hits.

## Supplementary Materials

The following are available online at https://www.mdpi.com/1422-0067/21/8/2861/s1, Table S1. Full LC-MS/MS analysis of F95-enriched proteins in bovine serum. Supplementary Table S2. Full LC-MS/MS analysis of F95-enriched proteins in bovine serum-EVs.

## Abbreviations

BSA	Bovine serum albumin
CD63	CD63 antigen; granulophysin; lysosomal-associated membrane protein 3
CoV	Coronavirus
COVID-19	Coronavirus disease 2019
ECL	Enhanced chemiluminescence
ECM	Extracellular matrix
EVs	Extracellular vesicles
F95	Pan-deimination/citrullination antibody
FBS	Fetal bovine serum
Flot-1	Flotillin-1
HIF	Hypoxia inducible factor
Ig	Immunoglobulin
KEGG	Kyoto encyclopedia of genes and genomes
LC-MS/MS	Liquid chromatography mass spectrometry
NETosis	Neutrophil extracellular trap formation
NTA	Nanoparticle tracking analysis
PAD	Peptidylarginine deiminase
RAP1	Ras-related protein 1
SARS	Severe acute respiratory syndrome
SDS-PAGE	Sodium dodecyl sulfate polyacrylamide gel electrophoresis
TBS	Tris buffered saline
TEM	Transmission electron microscopy
WB	Western blotting
